# Novel Therapeutic Strategies and Biomarkers in the Management of Inflammatory Bowel Disease: A Narrative Review

**DOI:** 10.7759/cureus.113698

**Published:** 2026-07-30

**Authors:** Zechariah Kandathil, Arif Raihan, Thivian Mathanaruban

**Affiliations:** 1 Geriatrics, Ashford and St. Peter's Hospitals NHS Foundation Trust, Chertsey, GBR; 2 Paediatrics, Dartford and Gravesham NHS Trust, Dartford, GBR; 3 General Surgery, West Hertfordshire Teaching Hospitals NHS Trust, Watford, GBR

**Keywords:** biomarkers, crohn’s disease (cd), inflammatory bowel diseases (ibd), janus kinase, novel treatments, ulcerative colitis (uc)

## Abstract

Inflammatory bowel disease (IBD) is a multitude of chronic inflammatory conditions that affect the gastrointestinal (GI) tract. These disorders, which primarily include Crohn’s disease (CD) and ulcerative colitis (UC), can significantly impact an individual's quality of life due to their persistent and usually unpredictable presentations. The current biomarkers and treatments that are available have their limitations, and therefore, it is important that novel treatments and new biomarkers are readily being researched in order to help improve the quality of life of patients as well as to reduce the financial burden of treatment on the National Health Service (NHS). This essay will look into the biomarkers and novel treatments used in the diagnosis and management of IBD in adult patients.

## Introduction and background

Inflammatory bowel disease (IBD) comprises a group of chronic inflammatory conditions affecting the gastrointestinal (GI) tract, primarily Crohn's disease (CD) and ulcerative colitis (UC) [1]. Both conditions substantially impair quality of life owing to their persistent and unpredictable course. Existing biomarkers and treatments each carry important limitations, which have driven ongoing research into novel biomarkers and therapies aimed at improving patient outcomes and reducing the financial burden of care on health systems such as the National Health Service (NHS). This narrative review summarises the pathophysiology and clinical features of CD and UC, appraises established and emerging biomarkers used in diagnosis and monitoring, and reviews novel therapeutic classes, including Janus kinase (JAK) inhibitors, sphingosine-1-phosphate receptor (S1PR) modulators, and selective interleukin-23 (IL-23) antagonists, in the management of adult IBD. The review also outlines its search and selection approach and its limitations as a narrative synthesis, in line with recommendations for transparent reporting of literature reviews.

Crohn's disease

CD is a lifelong inflammatory condition that may affect any part of the GI tract, from mouth to anus, most commonly the terminal ileum and colon [2]. The disease burden is not evenly distributed: approximately 80% of patients have small bowel involvement, of whom around 30% have ileitis exclusively; 50% have ileocolitis, 20% have colitis exclusively, and 30% develop perianal disease [3]. Inflammation is discontinuous, producing characteristic 'skip lesions', and extends transmurally through the full thickness of the bowel wall to the serosa. This transmural pattern predisposes patients to deep ulceration and complications, including strictures (luminal narrowing) and fistulae (abnormal connections between bowel segments or with other organs) [4]. Diagnosis requires a composite assessment incorporating clinical history, blood and faecal tests, endoscopy, and imaging. Management aims to induce and maintain remission and optimise quality of life through corticosteroids, aminosalicylates, biologic agents, dietary and lifestyle modifications, and, in complicated disease, surgery.

Pathophysiology

The precise aetiology of CD remains unknown, but current evidence points to a cascade beginning with epithelial barrier dysfunction [5]. The intestinal epithelium normally maintains gut homeostasis and excludes harmful luminal contents [6]; in CD, increased permeability permits bacterial translocation. Antigen-presenting cells then recognise microbial antigens as foreign, triggering an immune cascade [7] with the release of pro-inflammatory cytokines, including interleukin-1 (IL-1), IL-6, and tumour necrosis factor-alpha (TNF-α), which recruit neutrophils and macrophages to the site of inflammation, amplifying tissue damage [8].

A subset of patients develop granulomas, aggregations of immune cells that contribute to chronic tissue damage and aid histological diagnosis [9]. Unlike UC, CD is characterised by transmural rather than mucosa-limited inflammation [10], interspersed with skip lesions of unaffected tissue [4,10], which together contribute to the varied complications, including strictures, fistulae, and abscesses, seen across the GI tract [10]. Extraintestinal manifestations affecting the skin, eyes, and joints may also occur, reflecting the systemic nature of the inflammatory response [11].

Clinical Presentation

CD typically first presents in late adolescence or early adulthood, often with non-specific symptoms such as lethargy, fatigue, and diarrhoea. Abdominal pain varies in location depending on the affected segment; terminal ileal inflammation may mimic appendicitis with right lower quadrant pain. Diarrhoea is often non-bloody, though blood or mucus suggests more severe ulceration [12]. Weight loss and malnutrition arise from malabsorption, anorexia, and protein-losing enteropathy, the latter contributing to hypoalbuminaemia and peripheral oedema [13]. Perianal disease may manifest as fissures, abscesses, and complex fistulae requiring surgical intervention [14]. Oral manifestations (aphthous ulcers and cobblestoning) are rarer but informative, and obstructive symptoms from strictures or adhesions may necessitate surgical management. The cause of CD remains incompletely understood, but genetic predisposition, environmental exposures, and immune dysregulation are all thought to contribute, with family history conferring the greatest increase in risk.

Ulcerative colitis

UC affects the colon and rectum, with inflammation typically confined to the innermost (mucosal) lining. Continuous inflammation extending proximally from the rectum leads to ulceration and symptoms, including bloody diarrhoea, abdominal pain, and urgency. Unlike CD, which may affect any part of the GI tract, UC is confined to the large intestine, and the inflammation tends to be more uniform and continuous rather than patchy.

As with CD, the cause of UC is multifactorial, reflecting genetic, environmental, and immune contributions. Up to 15.5% of patients have a first-degree relative with the disease, underscoring the genetic contribution [15]. Environmental factors appear particularly important in developed countries, potentially reflecting improved hygiene and sanitation and consequently reduced childhood exposure to enteric infection, which may impair maturation of the mucosal immune system and predispose to an inappropriate immune response on later exposure. Prior GI infection has also been proposed to alter the gut flora and trigger chronic inflammation in genetically susceptible individuals [15].

Pathophysiology

In UC, the epithelial barrier is compromised by reduced synthesis and altered sulphation of mucin 2, with inflammation limited to the mucosa and superficial submucosa (in contrast to the transmural involvement of CD). Increased permeability allows lymphocyte infiltration of the lamina propria and epithelium, while neutrophil infiltration produces crypt abscesses, collections of neutrophils within the crypt lumen that are characteristic of active disease. Mucosal ulceration is followed by granulation tissue formation, comprising proliferating fibroblasts, inflammatory cells, and new blood vessels, which may support healing. Depletion of mucus-producing goblet cells further compromises barrier function and perpetuates inflammation [15].

Classification

UC is classified according to the Montreal Classification, based on the anatomical extent of colonic involvement: ulcerative proctitis, in which inflammation is limited to the rectum; proctosigmoiditis, involving inflammation of the rectum and sigmoid colon; left-sided colitis, in which inflammation extends from the rectum to the splenic flexure; pancolitis, in which inflammation affects the entire colon [15].

Clinical Features and Complications

Presentation varies with disease extent and severity, but common features include diarrhoea with blood, mucus, or pus (worsening during flares); crampy left lower quadrant pain relieved by defecation; rectal bleeding; urgency; and tenesmus. Weight loss may occur secondary to malabsorption, reduced appetite, or fear of eating, and fatigue is common [15].

Complications include toxic megacolon, an acute, severe colonic dilatation associated with systemic toxicity and a risk of perforation, sepsis, and peritonitis, as well as, in long-standing disease, an increased risk of colorectal cancer, necessitating regular surveillance colonoscopy [15].

## Review

Methods

Search Strategy

A literature search was conducted across PubMed/MEDLINE, Embase, and the Cochrane Library for articles published between 2000 and 2024, with an emphasis on evidence published within the preceding 10 years given the pace of change in IBD therapeutics. Search terms combined controlled vocabulary and free-text keywords for the disease ("inflammatory bowel disease," "Crohn's disease," "ulcerative colitis") with terms relating to diagnosis and monitoring ("biomarker," "faecal calprotectin," "C-reactive protein," "faecal immunochemical test," "leucine-rich alpha-2 glycoprotein," "prostaglandin E metabolite"), and treatment ("biologic," "Janus kinase inhibitor," "sphingosine-1-phosphate receptor modulator," "interleukin-23 antagonist," named drug terms), combined using the Boolean operators AND/OR. Reference lists of retrieved articles and relevant reviews were hand-searched to identify additional eligible studies.

Eligibility Criteria

Studies were considered eligible if they (i) involved adult (≥18 years) patients with CD or UC or in vitro/mechanistic work directly relevant to their pathophysiology; (ii) were published in English; (iii) reported on a biomarker or therapeutic agent relevant to the diagnosis, monitoring, or management of IBD; and (iv) were peer-reviewed primary research articles, meta-analyses, systematic reviews, or clinical guidance from a recognised professional body (e.g. the European Crohn's and Colitis Organisation). Studies were excluded if they concerned exclusively paediatric populations, were not available in full text, or reported solely on conditions other than CD or UC without direct relevance to IBD pathophysiology, biomarkers, or treatment.

Study Selection

Records identified through the search were screened by title and abstract, followed by full-text assessment of potentially eligible articles against the criteria above.

Data Extraction and Risk of Bias

For each included study, the following were extracted where reported: study design, population and sample size, intervention or biomarker under investigation, comparator, outcome measures, and key results (including point estimates and, where available, measures of precision). Given the narrative (rather than systematic) design of this review, a formal, study-by-study risk-of-bias assessment using a validated tool (e.g. the Cochrane Risk of Bias 2 tool for randomised trials, or ROBINS-I for non-randomised studies) was not performed. Instead, the evidence hierarchy was considered qualitatively when weighing findings: randomised controlled trials and meta-analyses were prioritised over observational cohort studies, and these, in turn, were prioritised over case series or expert opinion. This is noted in the text where relevant (e.g. in relation to conflicting findings on faecal calprotectin (FCP) performance in CD versus UC). This represents an important limitation relative to a full systematic review and is addressed further in the "Discussion and Limitations" section.

Statistical Approach

This review is a narrative synthesis and does not include an original meta-analysis or meta-regression. Where possible, results are reported using point estimates (e.g. percentages achieving remission or response) and, where available in the source publication, p-values or 95% confidence intervals (CIs), as originally reported by the primary studies. These are not independently pooled or re-analysed here. Where a cited source did not report a confidence interval alongside a point estimate or p-value, this is noted rather than a CI being supplied. Given the absence of a pooled quantitative analysis, formal statistical peer review is not considered essential for this review; however, editorial oversight of the accuracy of transcribed statistics from primary sources is recommended, and a statistician's input would be needed if the review were converted into a formal meta-analysis in the future.

Diagnostic approaches: endoscopy and imaging

The gold-standard test for diagnosing IBD is ileocolonoscopy, though this is costly, invasive, and time-consuming [16]. Endoscopy and imaging are central to diagnosis, to distinguishing CD from UC, and to guiding management [17] and are used both in the initial assessment of suspected IBD and for patients with confirmed disease. Endoscopy's principal advantage is the direct visualisation of the bowel, allowing assessment of disease severity and monitoring of its evolution over time. Ileocolonoscopy also allows biopsy sampling. Because of its invasiveness and cost, it is used less frequently for ongoing disease-activity monitoring.

Less invasive endoscopic techniques have since been developed, including single- or double-balloon-assisted enteroscopy (SBE/DBE), video capsule endoscopy (VCE), and confocal laser endomicroscopy (CLE). VCE uses a swallowed wireless capsule to image the whole gut, including regions inaccessible to colonoscopy, and is markedly less invasive and more cost-effective than ileocolonoscopy, although capsule retention remains a concern for some patients and biopsy is not possible [18-20]. CLE permits microscopic imaging of live tissue during colonoscopy, allowing more rapid diagnosis [21], while enteroscopy permits access to small intestinal regions inaccessible to conventional endoscopy and allows histological sampling, though its technical complexity and lengthy preparation make it unsuitable for initial assessment [22]. Because imaging techniques, while precise, are limited by cost, workforce availability, and equipment requirements, a reliable biomarker offering standardised, rapid, and affordable testing would be of substantial clinical value [18-22].

Biomarkers in IBD

Established Biomarkers

Faecal calprotectin: It is the biomarker most widely used in IBD diagnosis and management. It is a calcium-binding protein composed of S100A8 and S100A9 [23] and is derived predominantly from neutrophils, where it contributes to the innate immune response and has direct antimicrobial activity [24]. In IBD, FCP reflects granulocyte migration through the intestinal wall [25] and, as a single biomarker, provides the strongest evidence for active disease in clinical practice.

Evidence on its comparative performance in CD versus UC is conflicting: one meta-analysis suggested that FCP may better reflect disease activity in UC than CD [26], while other studies have found the reverse [27]. Further research is needed to resolve this discrepancy. FCP reduction with treatment has been associated with a reduced relapse risk [28], although it may take up to two weeks for FCP to fall despite earlier symptomatic improvement, limiting its use for assessing the very short-term treatment response. The European Crohn's and Colitis Organisation recommends monitoring FCP every three months when levels are negative and monthly when levels are elevated [29]. Interpretation must account for confounders: FCP is reduced in pregnancy (risking false negatives if unadjusted) and raised by proton pump inhibitors, non-steroidal anti-inflammatory drugs (NSAIDs), coeliac disease, colorectal cancer, diverticulitis, intestinal infection, and non-pathological factors such as obesity, age, and lifestyle [30].

C-reactive protein: C-reactive protein (CRP) is synthesised in the liver under IL-6 stimulation and rises with inflammation [31]. It is useful for assessing disease activity and treatment efficacy, but its lack of specificity limits its diagnostic value [32] because CRP also rises in non-GI inflammatory conditions, diabetes, cardiovascular disease, and malignancy. Although CRP is typically higher in CD than in UC, it cannot reliably distinguish between the two [33].

CRP normalisation appears prognostically useful: normalisation at 14 weeks after starting infliximab was associated with a greater likelihood of remaining in remission at one year [34], and higher baseline CRP has been associated with a worse response to infliximab [35] and, over a four-year follow-up, to adalimumab [36]. Several studies measuring CRP at intervals of six weeks to three months have found higher CRP predictive of relapse or recurrence [37,38], although this is not a universal finding: one study found no correlation between CRP and endoscopic scores 54 weeks after surgery [39]. Taken together, CRP appears useful for monitoring treatment response and predicting relapse in aggregate, but conflicting individual study findings warrant a degree of caution in overinterpreting any single result.

Faecal immunochemical test: The faecal immunochemical test (FIT) measures faecal haemoglobin concentration using an antibody specific to human haemoglobin. Although developed primarily for colorectal cancer screening, it may also help detect mucosal lesions in IBD: one study identified IBD in 35 of 236,000 asymptomatic patients who tested FIT-positive during colorectal cancer screening [40]. However, FIT can also be positive due to unrelated causes such as haemorrhoids, and its sensitivity is lower for right-sided or proximal disease than for distal colonic disease, which may limit its usefulness in UC recurrence localised to the right colon [41].

Novel Biomarkers

Leucine-rich alpha-2 glycoprotein: Leucine-rich alpha-2 glycoprotein (LRG) is a glycoprotein containing eight leucine-rich domains [42], expressed by neutrophils, hepatocytes, macrophages, and intestinal epithelial cells and induced by cytokines, including TNF-α, IL-22, and IL-1β [43,44]. LRG gene expression is increased in peripheral blood mononuclear cells in IBD [46] and is thought to reflect intestinal inflammation more directly than serum CRP because cytokine-stimulated neutrophils and inflamed epithelium both contribute to its production. LRG has been shown to be elevated in active IBD even when CRP is normal [43].

Although, LRG has been shown to be elevated in active IBD and even lower in UC patients who have achieved histological healing, LRG is not IBD-specific and is also elevated in diabetes, gastric and colorectal cancer, psoriasis, heart failure, and obesity [46-52]. Some studies have found no significant difference between UC patients and healthy controls [53], and others suggest that LRG performs comparably to, rather than better than, CRP, FIT, and FCP [54]. LRG may nonetheless have a future role, particularly in monitoring patients with low disease activity, but it is not currently established as superior to existing biomarkers.

Prostaglandin E-major urinary metabolite: Prostaglandin E-major urinary metabolite (PUM) is released into the circulation from areas of active inflammation; because of its short plasma half-life, urinary measurement has been proposed instead [55,56]. It is a non-specific marker, also raised by smoking, malignancy, and laxative use [57-60]. UC activity correlates closely with PUM levels, which have also been shown to predict histological remission [61], reflect endoscopic activity in long-standing UC [62], and predict recurrence more accurately than endoscopic scoring in one study [63]. PUM shows considerable future promise in UC monitoring, although its lack of disease specificity means that further validation is required before wider clinical adoption.

An ideal IBD biomarker would be non-invasive, cost-effective, and disease-specific; however, no biomarker currently available meets all three criteria, meaning endoscopy remains the diagnostic gold standard. Table 1 summarises the biomarkers discussed above.

**Table 1:** Biomarker comparison. FCP: faecal calprotectin; CRP: C-reactive protein; FIT: faecal immunochemical test; LRG: leucine-rich α2-glycoprotein; PUM: prostaglandin E-major urinary metabolite; IL: interleukin; GI: gastrointestinal; CD: Crohn’s disease; CRC: colorectal cancer; UC: ulcerative colitis; PPIs: proton pump inhibitors; NSAIDs: non-steroidal anti-inflammatory drugs.

Biomarker	Biological source	Principal clinical use	Key limitations
FCP	Neutrophil-derived (S100A8/A9)	Most widely used marker of mucosal inflammation; used for monitoring activity and relapse risk	Confounded by pregnancy, PPIs, NSAIDs, other GI pathology, obesity, and age; lag of up to two weeks after treatment change
CRP	Hepatic synthesis (IL-6 driven)	Assessing inflammation and treatment response; prognostic for relapse	Non-specific; poor discrimination between CD and UC; can remain normal in active disease
FIT	Faecal haemoglobin (antibody-based)	Opportunistic detection of mucosal lesions; primarily a CRC screening tool	False positives (e.g. haemorrhoids); reduced sensitivity for proximal/right-sided disease
LRG	Neutrophils, hepatocytes, macrophages, and intestinal epithelium	Reflecting mucosal inflammation and histological healing in UC	Elevated in numerous non-IBD conditions; not shown to be superior to CRP/FCP/FIT
PUM	Released from sites of inflammation; measured in urine	Correlates with UC activity, histological/endoscopic status, and recurrence risk	Non-specific (smoking, malignancy, and laxatives); requires urinary sampling due to short plasma half-life

Novel therapeutic strategies

IBD typically follows a relapsing-remitting course requiring lifelong treatment, historically managed with corticosteroids, aminosalicylates, and immunosuppressants [64]. While these have improved prognosis and quality of life through remission induction and complication prevention, up to half of patients experience either loss of response to biologics or primary non-response. This, together with a shift in treatment goals from symptom control towards mucosal healing, underpins the need for novel therapies. Additional drivers include the infection and malignancy risks of long-term immunosuppression, particularly relevant in a post-pandemic context [65]; the potential for biosimilars to reduce cost while preserving efficacy [66]; and the burden that intravenous administration places on both health services and patients' social and working lives. Oral or subcutaneous alternatives may improve adherence [65]. Several novel therapeutic classes are discussed below.

Janus Kinase Inhibitors

Janus kinase inhibitors (JAK-i) target cytoplasmic non-receptor tyrosine kinases within the JAK family (JAK1, JAK2, JAK3, and TYK2) [67]. JAKs transduce cytokine and growth factor signals and are central to inflammation in autoimmune disease, including IBD [68]. Cytokine signalling occurs via JAK-mediated phosphorylation and dimerisation of signal transducer and activator of transcription (STAT) proteins, which then translocate to the nucleus and initiate transcription of immune and inflammatory genes [69]. Because small-molecule JAK-i can target multiple cytokine-dependent pathways simultaneously, rather than a single cytokine as with earlier biologics, they represent a potentially significant advance in IBD treatment [70]. Tofacitinib and filgotinib are approved for UC, and upadacitinib is approved for both UC and CD [71,72].

Tofacitinib: Tofacitinib, the first approved JAK-i, is used in moderate-to-severe UC. It preferentially inhibits JAK1 and JAK3, blocking signalling of IL-2, IL-3, IL-4, IL-5, IL-6, IL-12, IL-15, IL-21, and interferon-gamma [73-75]. Real-world data show that 74% of patients achieve clinical response by eight weeks and 44% achieve corticosteroid-free remission by week 26 [76]. Efficacy is not universal; however, younger age at initiation and higher baseline CRP have been associated with primary non-response [76], and overall, the evidence on efficacy remains mixed, warranting further research. In the OCTAVE trials, adverse events were less frequent than placebo at the 5 mg dose but more frequent at the 10 mg dose; the most common adverse effects were headache, arthralgia, and nasopharyngitis [77].

Filgotinib: Filgotinib preferentially inhibits JAK1 [78] and is approved for moderate-to-severe UC on the basis of rapid onset of action and effective symptom relief, including maintenance of remission. Caution is advised in patients with risk factors including active smoking, prior malignancy, age over 65, and pre-existing cardiac disease [79]. Although studied in CD, filgotinib is not licensed for this indication following failure to meet endpoints in phase III trials, which are large controlled studies (typically involving hundreds of patients) that compare a new treatment with the current standard of care [80].

Upadacitinib: Upadacitinib, a JAK1 inhibitor, blocks signalling of IL-2, IL-4, IL-6, IL-7, IL-9, IL-15, IL-21, and interferon-gamma, cytokines central to CD pathogenesis [81]. It induced clinical remission in a greater proportion of CD patients than placebo across all doses tested; endoscopic remission was achieved in 8-22% of patients on active treatment versus 0% on placebo, with the greatest clinical and endoscopic benefit seen at 24 mg twice daily [82,83]. Data from rheumatoid arthritis, psoriatic arthritis, and atopic dermatitis populations suggest a higher risk of serious infection at 30 mg compared with lower doses; findings on overall adverse event rates versus placebo have been mixed, with 45 mg associated with more adverse events than placebo in some studies. The most common adverse effects were acne, nasopharyngitis, and elevated creatine phosphokinase; the most common serious adverse effects were herpes zoster infection, neutropenia, and lymphopenia [82].

Sphingosine-1-Phosphate Receptor Modulators

Sphingosine-1-phosphate (S1P) signals through cell-surface G-protein-coupled receptors S1P1-S1P5. S1P1-3 are widely expressed throughout the body, while S1P4-5 are more restricted to lymphoid, haematopoietic, and central nervous system tissues. S1PR modulators act by trapping lymphocytes within lymphoid organs, preventing their trafficking to sites of intestinal inflammation [83].

Ozanimod: Ozanimod is an oral agent selective for S1PR1 and S1PR5, receptors central to lymphocyte trafficking in IBD pathogenesis [84]. In a study of a 1 mg daily dose as induction and maintenance therapy, 18.4% of patients achieved clinical remission by week 10 versus 6% on placebo, with clinical response seen in 47.8% versus 25.9%, alongside greater endoscopic improvement and mucosal healing. Serious infection occurred in under 2% of patients regardless of dose; transaminase elevation was observed but without drug-induced liver injury [85]. Because ozanimod can cause atrioventricular conduction delay and transient bradycardia, baseline ECG is recommended; varicella zoster virus (VZV) antibody testing and vaccination (where seronegative) are advised before initiation given a risk of VZV meningitis, and ophthalmic evaluation is recommended in patients with diabetes or uveitis owing to the risk of macular oedema [86].

Etrasimod: Etrasimod, an oral agent selective for S1PR1, S1PR4, and S1PR5, has been studied in patients with moderate-to-severe active UC. In a trial comparing 1 mg and 2 mg doses with placebo over 12 weeks using the modified Mayo Clinic Score (stool frequency, rectal bleeding, and endoscopic findings), the 2 mg dose produced significantly greater improvement than placebo, with a higher proportion achieving endoscopic improvement. The safety profile was favourable overall, supporting its potential as an option for both induction and maintenance of remission [87,88].

Selective Interleukin-23 Antagonists

IL-12 and IL-23 share the p40 subunit, with IL-12 comprising p35/p40 and IL-23 comprising p19/p40, but mediate distinct inflammatory pathways: IL-23 drives Th17 differentiation and IL-17 upregulation, recruiting neutrophils, macrophages, and dendritic cells to the intestine, while IL-12 drives a Th1/TNF-mediated response [83,89-91]. Ustekinumab, targeting the shared p40 subunit, is already established for UC and CD.

Selective IL-23 antagonists instead target the unique p19 subunit, providing more selective inhibition of IL-23 signalling; examples include brazikumab, risankizumab, guselkumab, and mirikizumab, of which risankizumab and mirikizumab have the most robust evidence base [92].

Risankizumab: Risankizumab targets the IL-23 p19 subunit. CD patients show increased IL-23 p19 mRNA expression compared with healthy controls, and UC patients with high serum IL-23 have worse clinical outcomes; genetic studies have also linked IL-23 receptor polymorphisms to IBD susceptibility [92]. Risankizumab trials in CD were notable for being the first to co-primary clinical remission (via the CD Activity Index) and endoscopic response (via the Simple Endoscopic Score for CD), alongside secondary endpoints including FCP, endoscopic remission, high-sensitivity CRP, and IL-22 [92].

In the FORTIFY maintenance trial (462 patients), participants received subcutaneous risankizumab 180 mg or 360 mg every eight weeks or were withdrawn to placebo; non-responders could receive open-label intravenous risankizumab 1200 mg. Both maintenance doses met the co-primary endpoints of clinical remission and endoscopic response at 52 weeks, with numerically greater efficacy for the 360 mg dose across several secondary measures, including a numerically higher rate of endoscopic remission, although adverse event rates were similar between doses. Patients who discontinued risankizumab after intravenous induction showed persistent clinical remission at week 52 but did not maintain endoscopic response, suggesting a possible dissociation between clinical and endoscopic durability that warrants further study, as do optimal maintenance dosing and the timing of rescue therapy [91].

Mirikizumab: Mirikizumab has been evaluated in moderate-to-severely active UC through paired induction (intravenous administration every four weeks for 12 weeks) and maintenance (subcutaneous administration every four weeks for 40 weeks) trials, both with clinical remission as the primary endpoint. Both trials showed higher remission rates with mirikizumab than placebo [92]. The most common adverse effects were nasopharyngitis and arthralgia, occurring more frequently than with placebo. Opportunistic infections were reported in 15 patients, including herpes zoster in six and malignancy in eight patients on mirikizumab, compared with one case of herpes zoster and no malignancies on placebo, a numerical imbalance that warrants continued pharmacovigilance, although the trial was not powered to assess malignancy risk formally [73].

Table 2 summarises the mechanism of action, approved indications, and reported adverse effects of the agents discussed above.

**Table 2:** Novel therapeutic strategies. JAK: Janus kinase; IL: interleukin; CD: Crohn’s disease; UC: ulcerative colitis; VZV: varicella zoster virus; IFN: interferon; LFT: liver function test; CPK: creatine phosphokinase.

Agent	Class	Mechanism of action	Approved indication (as discussed)	Commonly reported adverse effects
Tofacitinib	JAK inhibitor	JAK1/JAK3 inhibition; blocks IL-2, 3, 4, 5, 6, 12, 15, 21, and IFN-γ	Moderate-severe UC	Headache, arthralgia, nasopharyngitis (dose-dependent, higher at 10 mg)
Filgotinib	JAK inhibitor	Preferential JAK1 inhibition	Moderate-severe UC (not licensed for CD)	Caution in smokers, prior malignancy, age >65, cardiac disease
Upadacitinib	JAK inhibitor	JAK1 inhibition; blocks IL-2, 4, 6, 7, 9, 15, 21, and IFN-γ	UC and CD	Acne, nasopharyngitis, raised CPK; serious: herpes zoster, neutropenia, lymphopenia; higher infection risk at 30 mg
Ozanimod	S1PR modulator	Selective S1PR1/S1PR5 agonism/functional antagonism; sequesters lymphocytes in lymphoid organs	UC	AV conduction delay/bradycardia, VZV reactivation risk, macular oedema risk (diabetes/uveitis), transient LFT rise
Etrasimod	S1PR modulator	Selective S1PR1/S1PR4/S1PR5 modulation	UC (studied)	Generally favourable safety profile in trials to date
Ustekinumab	IL-12/23 antagonist (anti-p40)	Blocks shared p40 subunit of IL-12 and IL-23	UC and CD (established)	Standard biologic infection risk (not detailed in the reviewed sources)
Risankizumab	Selective IL-23 antagonist (anti-p19)	Selective inhibition of IL-23 signalling	CD	Not extensively detailed in the reviewed sources; standard biologic infection risk anticipated
Mirikizumab	Selective IL-23 antagonist (anti-p19)	Selective inhibition of IL-23 signalling	UC	Nasopharyngitis, arthralgia; opportunistic infection (including herpes zoster) and malignancy reported more often than placebo

Discussion and limitations

This review draws together evidence on the pathophysiology, biomarkers, and emerging therapeutics of IBD and highlights several important limitations that should be considered when interpreting its conclusions.

First, this is a narrative rather than a systematic review. While a search strategy and eligibility criteria are disclosed (Methods section), study identification and screening were not conducted by dual independent reviewers, no protocol was registered in advance (e.g. with PROSPERO), and risk of bias was considered only qualitatively rather than using a validated tool applied study-by-study. This limits the reproducibility of study selection and means publication or selection bias cannot be excluded.

Second, no meta-analysis or meta-regression was performed. Quantitative results reported here (percentages, p-values, and, where available, confidence intervals) reflect the original primary studies and have not been independently pooled, re-analysed, or adjusted for study quality or heterogeneity. Direct cross-study comparison of, for example, remission rates between different JAK inhibitors should therefore be made cautiously, as trial populations, endpoints, and follow-up durations differed.

Third, several biomarkers and agents (e.g. LRG, PUM, and risankizumab) have a comparatively limited evidence base relative to longer-established options, and conflicting findings, such as the disputed comparative performance of FCP in CD versus UC, remain unresolved and would benefit from adequately powered, prospective, head-to-head studies. These limitations do not undermine the value of the review as a synthesis of current understanding, but they do mean that it should be read as a foundation for further, more rigorous systematic evaluation rather than as a substitute for one.

## Conclusions

Considerable progress has been made in understanding and treating IBD, particularly in the development of targeted therapies and the discovery of novel biomarkers. Nonetheless, substantial challenges remain before IBD can be fully controlled or eradicated, warranting sustained and more comprehensive research. Biomarker discovery has already transformed diagnosis, enabling earlier detection, more precise treatment regimens, and a deeper understanding of underlying disease mechanisms. However, the complex interplay of genetic, environmental, and immunological factors in IBD means that currently available biomarkers still capture only part of a complicated clinical picture. Similarly, targeted therapies, including small-molecule inhibitors and biologics alike, have shifted the treatment paradigm towards more effective and better-tolerated options, expanding the pharmacological arsenal against IBD and offering the prospect of improved quality of life and disease control. Continued evaluation of long-term safety and individual variability in treatment response underscores the ongoing need for research into novel therapeutic approaches, and future work would benefit from a formal systematic review and meta-analysis to build on the narrative synthesis presented here.
